# Sex Differences in Foot and Ankle Sports Injury Rates in Elite Athletes: A Systematic Review and Meta-analysis of 25,687,866 Athlete Exposures

**DOI:** 10.1177/23259671251364261

**Published:** 2025-09-10

**Authors:** Adrian J. Talia, Nicholas A. Busuttil, Andrew Hotchen, Adrian R. Kendal, Rick Brown

**Affiliations:** * Department of Foot & Ankle Surgery, Nuffield Orthopaedic Centre, Oxford University Hospitals NHS Trust, Oxford, United Kingdom; ‡ Institute for Health & Sport, Victoria University, Melbourne, Victoria, Australia; Investigation performed at University of Oxford, Oxford, UK

**Keywords:** female sports injuries, foot and ankle injuries, football, risk factors, sex differences

## Abstract

**Background:**

Female sports participation is at an all-time high, from amateur to professional levels. There has been recent media and scientific focus on the higher rates of injury to the anterior cruciate ligament and head injuries in female athletes compared with male athletes. A similar association has not been emphasized in the foot and ankle. Hence, this research aims to establish the rate of foot and ankle injury at the professional level in female athletes compared with their male counterparts.

**Purpose/Hypothesis:**

The purpose of this study was to understand the rate of foot and ankle injuries in professional and semiprofessional athletes. It was hypothesized that female athletes are injured at higher rates compared with their male counterparts.

**Study Design:**

Systematic review; Level of evidence, 3.

**Methods:**

A systematic review was conducted using PRISMA (Preferred Reporting Items for Systematic Reviews and Meta-Analyses) guidelines. PubMed, Ovid EMBASE, and Ovid MEDLINE were searched for relevant papers up until October 23, 2023. Papers reporting on rates of foot and ankle injuries in female professional or semiprofessional athletes were included, along with a male comparison group. A total of 2510 papers were screened. A meta-analysis was performed on 4 separate subgroups using common and random-effects models.

**Results:**

A total of 53 papers met the inclusion criteria, and a meta-analysis of proportions was performed. Of this total, 21 records reported absolute elite athlete numbers and 32 reported athletic exposures. Meta-analyses were performed on these 2 subgroups separately. The literature was found to have a high risk of bias. The rate of injuries to the foot and ankle in female athletes was higher than their male counterparts overall (log odds ratio). Professional female athletes had significantly more injuries compared with their male counterparts using a common-effects model (odds ratio, 1.52 [1.44-1.61]) Chi-square testing demonstrated significant heterogeneity.

**Conclusion:**

This systematic review and meta-analysis demonstrated that female athletes suffer foot and ankle injuries at professional and semiprofessional competition levels at higher rates than their male counterparts. The literature on this topic is limited to large observational studies with significant risk of bias and heterogeneity. The current research provided an understanding of the significant effects of foot and ankle injury rates, detailing the increased exposures that are present in female semiprofessional and elite sports.

Sports injuries have been a subject of increasing research interest in the past 2 decades.^30^ The rise in popularity of female professional sports in recent years has seen a subsequent rise in injuries in female athletes. Female athletes participating in team sports have reported higher rates of injury compared with their male counterparts.^23^ This can result in time away from the sport, high medical costs, a reduction in team performance, and chronic physical and mental health issues.^29,53,96^ Recent meta-analyses indicate that ankle sprains, concussion, and anterior cruciate ligament injuries occur at higher rates in female athletes.^24,60,70^ There may be anatomic,^19,100^ hormonal,^71^ and biomechanical reasons^28,59^ for these differences in injury rate.^49,80^ It is also postulated that there are cultural differences between the sexes that may contribute to this difference, with girls participating in less physical activity than boys during formative years.^92^ With specific reference to the foot and ankle, there are attempts to elucidate reasons for differences in injury rate comparing women to men with ankle ligamentous injury^28^ and to elucidate anatomical differences in osteology.^63^

In recent years, female professional sporting competitions have increased in size, number, and popularity^28,69^ and have generated an increased media interest for women’s professional sports.^2^ This has a flow-on effect for amateur competitions, both junior and adult, resulting in increased numbers of injuries presenting to emergency departments and acute clinics.^45,72^ The bulk of literature thus far has concentrated on female knee injuries, specifically anterior cruciate injury,^4^ as well as head injuries, specifically concussion.^89^ Foot and ankle injuries have not been reported on to the same extent^31^ and it is difficult to paint a summative picture of sex-specific foot and ankle difference in injury rates from the current literature.^31,86^ It is even more difficult to find data on foot and ankle injuries in professional female athletes because most of the current available evidence stems from junior, high school, or collegiate athletes.^24,51,68^ The aim of this research is to examine, based on the current evidence available, the rate of foot and ankle injuries in the professional and semiprofessional settings. Our hypothesis is that female athletes are injured at higher rates than their male counterparts.

## Methods

This study focuses on professional and semiprofessional female athletes, because the quality of data collection and both injury surveillance and reporting is more complete than for amateur athletes.^8,9^ A systematic literature search was performed according to the most recent PRISMA (Preferred Reporting Items for Systematic Reviews and Meta-Analyses) guidelines.^5,66,91^ Data extraction, synthesis, and meta-analysis were performed according to the guidelines prescribed by the Meta-analysis of Observational Studies in Epidemiology group.^88^ The review was prospectively registered with PROSPERO https://www.crd.york.ac.uk/prospero/ (CRD42023475089) on October 22, 2023.

### Literature Search Strategy

Electronic databases were searched from inception until October 23, 2023. We searched PubMed, Ovid EMBASE, and Ovid MEDLINE. We searched for the following keywords in the title and/or abstract: “injuries,” “trauma,” “fracture,” “sprain,” “traumatic,” “acute,” “foot,” “ankle,” “toe,” “lower extremity,” “Lisfranc,” “metatarsal,” “tarsal,” “ankle,” “hindfoot,” “heel,” “Achilles,” “sport,” “athlete,” “female,” “women,” “girl,” and “gender.” Exclusion terms included “review” and “case report.” We combined terms using Boolean operators where appropriate. Our comprehensive search strategy with database-specific search terms is detailed in Appendix Table A1.

### Inclusion and Exclusion Criteria

Screening criteria were decided upon a priori. The research question was framed in terms of the Population Intervention Comparison Outcomes Study design framework

- *Population:* female athletes competing at a professional, semiprofessional, or collegiate level
- *Intervention/exposure:* a documented injury, resulting from the athletic activity to the foot and/or ankle region
- *Comparison:* male athletes competing in the same, or sex-comparable, sport at the same level, published in the same study
- *Outcome:* rate of injury of female athletes, as compared with their male counterparts
- *Additional screening criteria:* inclusion criteria: full text available, clinical study, injury rates reported, female athletic injuries reported *with* a male athlete comparison group, English-language study, human study, foot and ankle injuries reported, semiprofessional (eg, collegiate) or professional athletes. Exclusion criteria: pediatric patients, amateur athletes, review article, case report, nonclinical study, non–foot and ankle injuries, combat sports (eg, taekwondo or judo)

### Article Screening and Study Appraisal

**Figure 1.**
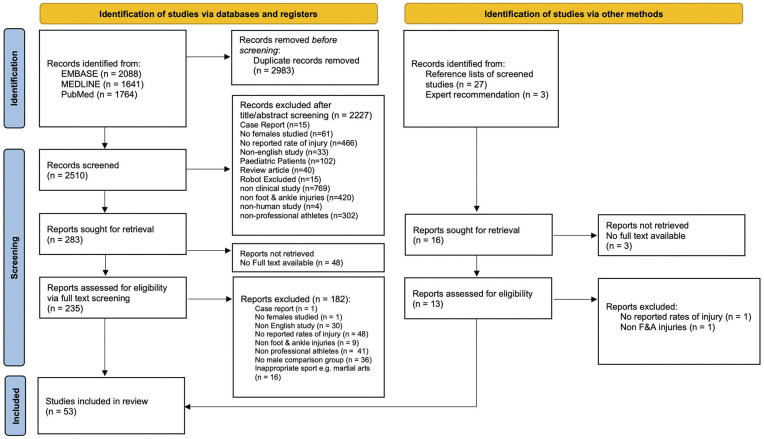
PRISMA 2020 (Preferred Reporting Items for Systematic Reviews and Meta-Analyses) flowchart. Adapted from Page MJ, McKenzie JE, Bossuyt PM, et al. The PRISMA 2020 statement: an updated guideline for reporting systematic reviews. *BMJ.* 2021;372:N71.^66^ doi:10.1136/bmj.n71. For more information, visit: http://www.prisma-statement.org/. F&A, foot and ankle.

We employed 2-pass screening. First, all duplicates were removed. Then each article title and/or abstract was screened independently by 2 authors (A.J.T. and N.A.B.) according to the above screening criteria. Of the remaining suitable articles, full text was then screened to determine final suitability. Any disagreements were resolved by the senior author (R.B.). The PRISMA flowchart is depicted in Figure 1 and details the steps taken to arrive at the final included articles. The Cochrane Risk Of Bias in Non-Randomized Studies of Exposures (ROBINS-E) tool^34^ was used to assess risk of bias in each of the articles, and the *robvis* online tool^58,74^ was used to provide graphical representation of this assessment. These are freely available, validated online tools tailored for population-based epidemiological studies.

### Definitions

The final papers included in the analysis reported injury rates either as a proportion of total athletes or in terms of athletic exposures (AEs); as defined in previous research.^42,43,43^ A reportable AE is defined as 1 athlete participating in 1 practice or competition, in which that athlete was exposed to the possibility of athletic injury. A reportable injury was defined as an injury that (1) occurred as the result of participating in a sanctioned practice or competition and (2) that required attention from a trainer or physician.

### Data Collection and Statistical Analysis

Data were then collected and recorded on all studies that met the inclusion criteria. Variables collected included the sport, level of competition, numbers of male and female participants or AEs, and numbers of foot and ankle injuries sustained by both male and female athletes. The reported incidence of injury was extracted if reported in the paper. Data were extracted from the main text, tables, or figures. Where appropriate, supplementary online material was used to locate the relevant data if it were not found in the main text.

### Injury Reporting

There was heterogeneity in the reporting of injury statistics. Where injury statistics were reported in terms of AEs, we extracted this as a preference, as it was a better representation of injury incidence. However, some papers reported on absolute numbers of athletes. If AE data were not available, then we extracted data on absolute athlete numbers. Any papers that did not report total numbers of athletes (ie, a sample size) were excluded from analysis.

#### Subgroup Analysis

The studies were then stratified into 4 groups depending on whether they reported absolute number of athletes, or AEs, and the level of competition. When there were multiple sports reported in a paper—for example, in papers reporting on American collegiate sports^13,15-18,50,76^—we extracted data from only sex-matched sports. For example, we did not extract data on male football (gridiron/American football) since there was no female equivalent.^39,46,75,79^

The outcome for analysis was injury rate of female athletes as a proportion of total female athletes. This was compared with a control group of male athletes reported in the same study. Data were tabulated using Microsoft Excel; and data analysis, including meta-analysis, was performed using the meta package Version 8.0-1 in R (Version 4.4.1; RStudio). A *P* value of <.05 was designated as the significance threshold.

### Meta-analysis

We performed 4 separate meta-analyses of the extracted data based on the defined subgroups. The reporting metric was the log odds ratio (OR) with athlete injury as a dichotomous outcome variable. A random-effects meta-analysis model, constructed using R, was the primary outcome due to expected exposure heterogenicity. There was heterogenicity in study populations due to participant ages, demographics, and different sports. Heterogeneity between studies was assessed using the *T*^2^ (study variance), the *I*^2^ (variability), and the maximum-likelihood estimator.^95^ The rank correlation test and the regression test, using the standard error of the observed outcomes as predictor, were used to check for funnel plot asymmetry.

## Results

### Literature Search

A total of 5493 studies were identified from database searches, and a further 27 from other sources (reference lists of included papers). 2983 duplicates were removed, leaving 2510 for an initial screening. 2227 were excluded based on title and abstract screening; 283 papers were included for full-text screening, of which 235 had full text available. After full-text screening, 53 studies (11 from grey literature, Figure 1) were deemed eligible for final inclusion (Figure 1). These studies report on a cumulative 25,687,866 AEs and 14,230 total athletes.

### Included Studies and Meta-analysis

A total of 53 studies were included in the analysis. In total, the 53 studies reported on 25,687,866 AEs (Appendix Table A2). Of these, 21 reported absolute athlete numbers (12 professional level, 9 semiprofessional level) and 32 reported athlete exposures (12 professional level, 20 semiprofessional level). Because the sample sizes (ie, the denominators) and competition levels were different in these subgroups, we performed separate meta-analyses for each. The complete extracted data used to perform analyses and references can be found in Appendix Table A2.

### Risk-of-Bias Assessment

**Figure 2.**
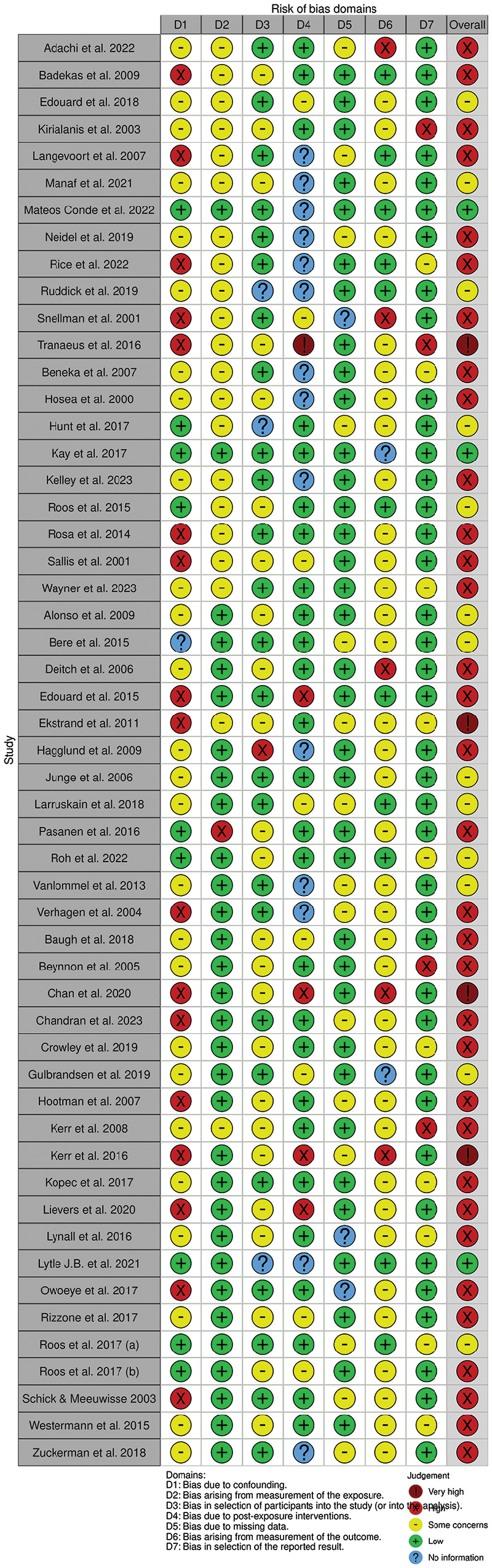
Individual study risk-of-bias assessment as determined by the Risk Of Bias In Non-randomized Studies of Exposures tool and visualized using robvis.

**Figure 3.**
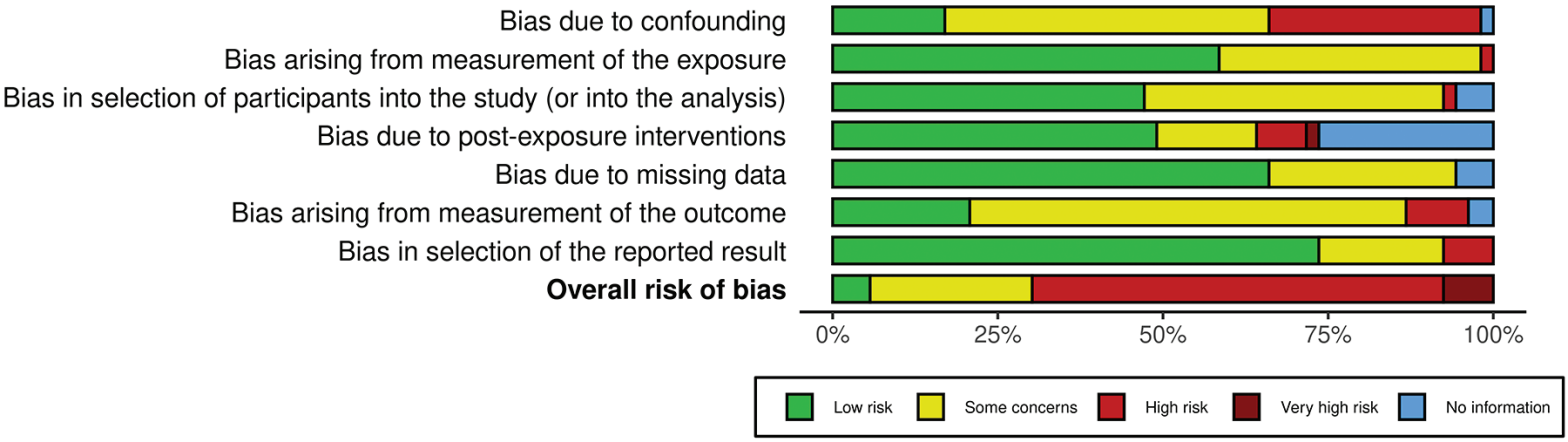
Summary graph demonstrating risk-of-bias assessment.

Figures 2 and 3 demonstrate the results of the risk-of-bias assessment using the ROBINS-E tool. Three of the included studies (5.7%) demonstrated a “low” risk of bias, 13 studies (24.5%) demonstrated “some concerns,” 33 studies had a “high” risk of bias (62.2%) and 4 studies (7.5%) were rated as having a “very high” risk of bias.

**Figure 4.**
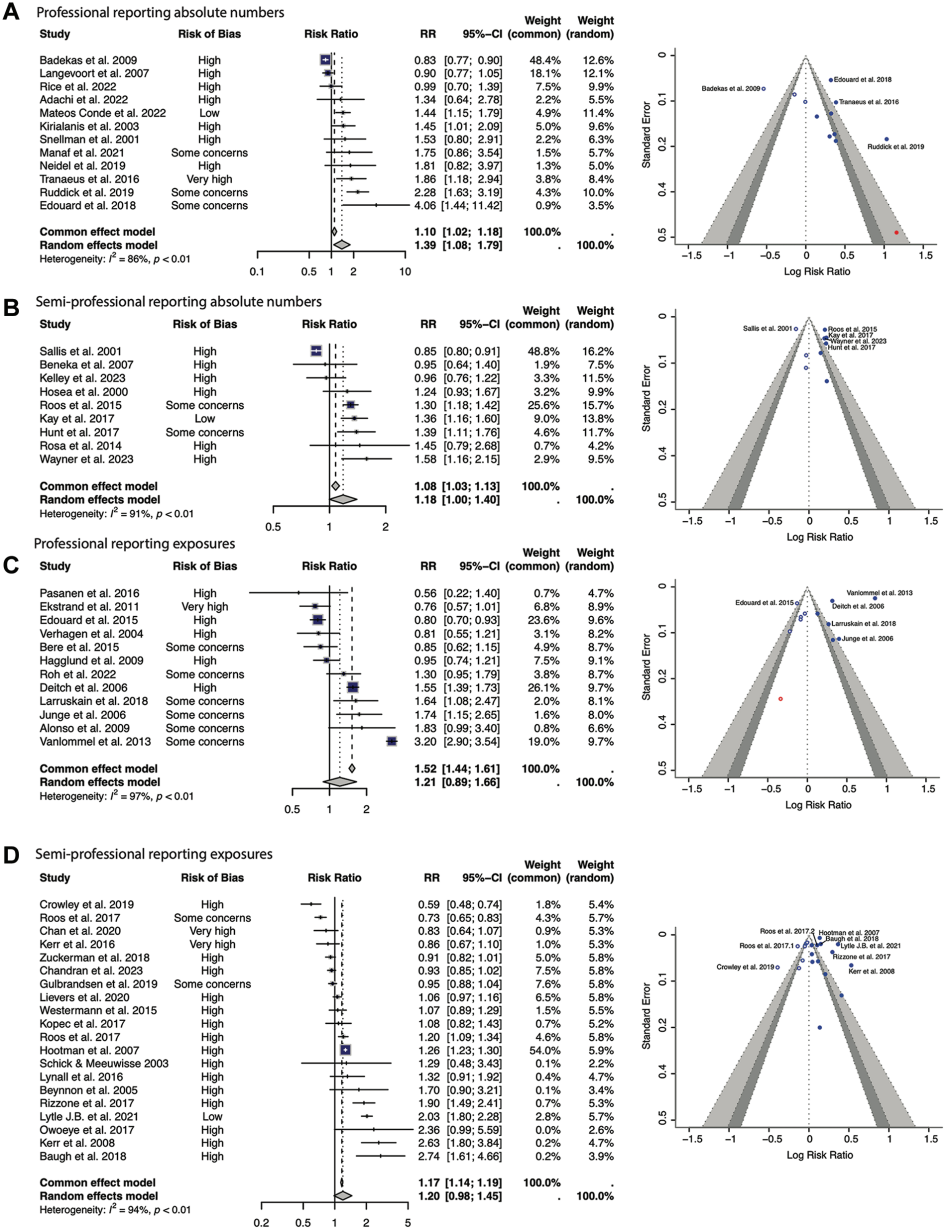
Meta-analysis results. Demonstrated are 4 forest plots with the corresponding funnel plots. Female athletes are represented on the right. (A) studies reporting absolute athlete numbers in professionals, (B) studies reporting absolute athlete numbers in semiprofessionals, (C) studies reporting athletic exposures in professionals, and (D) studies reporting athletic exposures in semiprofessionals. RR, risk ratio.

Figure 4 displays the meta-analysis for our 4 subgroups. We separately analyzed professional and semiprofessional athletes. In addition, we analyzed the studies reporting absolute numbers of athletes and AEs separately, due to the difference in the magnitude of numbers.

#### Studies Reporting Absolute Numbers of Athletes

##### Professional Athletes

A total of 12 studies were included in the analysis.^§^ There were 14,553 athletes in these studies when pooled, 7072 of whom were female. There was a significantly higher number of injuries in professional female athletes compared with male counterparts in the random-effects models (RR, 1.39; 95% CI, 1.08-1.79, respectively) (Figure 4A). One study had a low risk of bias (Mateos Conde et al),^57^ with the remainder scored as having some concerns of bias or high risk of bias (Figure 2). The *I*^2^ was 86% (*P* < .01) representing a significant degree of heterogenicity between study outcomes. The funnel plot demonstrates symmetry, with a wide effects range (Figure 4A).

##### Semiprofessional Athletes

Nine papers were included in the analysis.^‖^ There were 13,102 athletes in these studies when pooled, 6353 of whom were female. There was a significantly higher number of injuries in semiprofessional female athletes compared with male athletes using both common-effects (OR, 1.08; 95% CI, 1.03-1.13) and random-effects models (RR, 1.18; 95% CI, 1.00-1.40) (Figure 4B). One study had a low risk of bias,^39^ with the remainder having some concerns or high risk of bias (Figure 2). The *I*^2^ was 91% (*P* < .01) demonstrating heterogenicity between study outcomes. The funnel plot demonstrates asymmetry and a narrow effects range (Figure 4B).

#### Studies Reporting AEs

##### Professional Athletes

A total of 12 studies were included in the analysis.^¶^ This totaled 983,044 AEs, 323,313 of which were in female athletes. When measuring AEs, professional female athletes had significantly increased injuries compared with their male counterparts using a common-effects model (RR, 1.52; 95% CI, 1.44-1.61) but not in a random-effects model (RR, 1.21; 95% CI, 0.89-1.66) (Figure 4C). All studies had some concern or high risk of bias, with 1 study^27^ having a very high risk of bias (Figure 2). The study outcomes were significantly heterogeneous (*I*^2^ = 97%; *P* < .01). The funnel plot demonstrates symmetry and a narrow effects range (Figure 4C).

##### Semiprofessional Athletes

20 studies were included in the analysis.^#^ This totaled 43,856,776 AEs, 18,553,465 of which were in female athletes. Semiprofessional female athletes had a significantly increased number of injuries per AE compared with their male counterparts using a common-effects model (RR, 1.17; 95% CI, 1.14-1.19), but not using a random-effects model (RR, 1.20; 95% CI, 0.98-1.45) (Figure 4D). All studies had some concerns or high risk of bias, with 2 studies having a very high risk of bias (Figure 2).^14,43^ The outcomes reported high heterogeneity (*I*^2^ = 94%; *P* < .01). The funnel plot demonstrates symmetry and a wide effects range (Figure 4D).

## Discussion

Our meta-analysis of 53 studies totaling 25,687,866 AEs is the largest meta-analysis to our knowledge to examine sex differences in injury rates specific to foot and ankle injuries. Using a common-effects model meta-analysis, there was a significantly higher number of foot and ankle sporting injuries in professional (983,044 AEs: OR, 1.52; 95% CI, 1.44-1.61) and semiprofessional (43,856,776 AEs: OR, 1.17; 95% CI, 1.14-1.19) female athletes when compared with their male counterparts. Due to high risk of bias in many studies, there was a significant degree of heterogenicity between outcomes of included studies.

Most of the focus in the sports literature when comparing male and female injury rates has focused very broadly on injury rates,^13,16-18,50,76,99,103^ anterior cruciate ligament injuries,^20,102^ risk factors for injury,^20^ and concussion.^85^ Our finding of a higher injury risk in female athletes is in contrast to a recent meta-analysis by Zech and colleagues^102^ who reported a higher injury rate in male athletes; their review, however, was narrower in scope than our study and notably specifically excluded semiprofessional and American collegiate data, which made up a significant proportion of the included studies in this analysis.

Underlying reasons for the difference in injury rates have been examined. These include anatomic differences. Female feet have wider forefeet, shorter medial longitudinal arch, and shorter metatarsal length compared with male feet.^101^ Female foot and ankle osteology studies have demonstrated narrow canals and thinner bony cortices, which may place them at increased risk of stress fractures.^6^ Female athletes have a greater range of motion of the ankle, hindfoot, and midfoot while running, which may influence the type and severity of injuries sustained.^77^ Hormonal fluctuations may also place women at risk of injury via decreased ligamentous tensile strength, muscle recruitment, and neuromuscular control.^28^ Female athletes tend to exhibit increased ligamentous laxity, which when coupled with greater joint range of motion can place the joints in extreme positions, leaving them vulnerable to injury.^47^ In addition to these intrinsic reasons, there are extrinsic factors, such as the lack of female-specific footwear for certain sports,^62,64,97^ which may contribute to this observed difference in injury rates.

There were differences between different meta-analysis models that were used when assessing injuries sustained per AE compared with number of athletes who sustained injuries. When measuring AE, there was a significant difference between using a common-effects model but not using a random-effects model for both professional and semiprofessional athletes. We believe this is due to the breadth of the AE metric.

### Professional Versus Semiprofessional Athletes

We were interested in outcomes in different patient populations and so chose to analyze professional and semiprofessional athletes separately. Professional athletes are more likely to be full-time athletes with a team of professional support staff helping them achieve peak performance at the highest level (eg, Olympic Games or professional football). In contrast, the semiprofessional (eg, American collegiate) athlete, while still performing at an extremely high level, has other commitments or employment and will not have the same supports in place around him or her. Our analysis showed that, in both professional and semiprofessional athletes, female athletes experienced higher rates of foot and ankle injuries compared with male athletes. The magnitude of this difference was small: in professional athletes, the log ROR was 0.32, and in semiprofessional athletes, the RR was 0.18. This pattern suggests that the increased risk associated with being female may be a more influential factor than differences in the level of professional support available to athletes at different competition tiers. Importantly, the studies within both subgroups showed high variability, with I^2^ values exceeding 90%.

### Limitations

This is the first systematic literature search and meta-analysis, to our knowledge, to extract injury statistics from varied competition levels and different sports, and the first to report such a large number of AEs. This is only possible when extracting and pooling data from broader epidemiological studies and extracting foot and ankle–specific injuries. However, with such a broad scope of review, it is impossible to avoid heterogeneity in the data extracted. The presence of different sample sizes (absolute athlete numbers, AEs) means that we could not conduct a pooled meta-analysis of the 53 included papers, but had to divide our analyses into subgroups. The risk of bias evident on article review is a limitation of this study and is inherent to the literature, as these are observational studies of an exposure (ie, an injury).

The author group consists of 5 male researchers spanning 2 continents. There is a mix of clinicians and academics. There is a breadth of research disciplines (surgery, biostatistics, and biomechanics), including junior, midcareer, and senior researchers. The focus of this paper was on injury rates between sexes, and as such, a direct sex-based analysis is the primary objective of this research.

Many papers broadly report injury rates; foot and ankle injuries are not the specific focus of these papers, and many papers do not specify which injury was sustained. This could be addressed in future studies by narrowing the scope of the review. Data extraction to identify risk factors that would predispose to foot and ankle injury has been done previously by Collings et al,^20^ but this is beyond the scope of this study. The decision to focus on elite athletes may not be broadly applicable to most surgeons’ patient populations, but this is the subject of future research in our group. The decision to focus on elite athletes allows for some consistency in reporting, as they tend to have training and match play hours quantified and thus total AE is often a known quantity, but this is impossible in amateur athletes. Despite this, there is still significant heterogeneity in reporting injury incidence statistics, and this is reflected in our meta-analysis results.

## Conclusion

Our systematic literature review and pooled meta-analysis of 25,687,866 AEs demonstrates that across higher competitive levels and a range of sex-comparable sports, there is a higher injury incidence in female athletes compared with their male counterparts. The literature on this topic is limited to large observational studies with significant risk of bias and heterogeneity. With the issue of injury in women’s sports grabbing media attention, no doubt this is an expanding area of research. As further data become available and injury surveillance programs are carried out, more detailed analyses with more homogeneous data will be possible.

## Footnotes

§ References 1, 7, 26, 44, 48, 56, 57, 61, 73, 83, 87, 90.

‖ References 10, 36, 37, 39, 40, 80, 82, 84, 98.

¶ References 3, 11, 22, 25, 27, 33, 38, 49, 67, 78, 93, 94.

# References 9, 12, 14, 15, 21, 32, 35, 41, 43, 46, 52, 54, 55, 65, 75, 79, 81, 85, 99, 103.

## Appendix

**Appendix Table A1:** Literature Search Strategy

Databases searched using the following keywords seen in the title/abstract and/or abstract.
1. Injury
injuries OR injur* OR Trauma OR Fracture OR Sprain OR traumatic OR acute
2. Foot & Ankle
foot OR Ankle OR Toe OR lower extremity OR lower extremit* OR lower limb OR lower body OR Lisfranc* OR metatarsal* OR tarsal OR Ankle OR hindfoot OR heel OR achilles*
3. Sport-Related
Sport OR sport* OR athlete*
4. Female/Gender Specific
female OR women* OR girl* OR gender*
Exclude:
5. Case Reports and Literature Reviews
Title(“case report” OR review)
Final Search Plan:
1 AND 2 AND 3 AND 4 AND NOT 5
*Pubmed search:*
((((injuries[Title/abstract] OR injur*[Title/abstract] OR Trauma[Title/abstract] OR Fracture[Title/abstract] OR Sprain[Title/abstract] OR traumatic[Title/abstract] OR acute[Title/abstract]) AND (foot[Title/abstract] OR Ankle[Title/abstract] OR Toe[Title/abstract] OR lower extremity[Title/abstract] OR lower extremit*[Title/abstract] OR lower limb[Title/abstract] OR lower body[Title/abstract] OR Lisfranc*[Title/abstract] OR metatarsal*[Title/abstract] OR tarsal[Title/abstract] OR Ankle[Title/abstract] OR hindfoot[Title/abstract] OR heel[Title/abstract] OR achilles*[Title/abstract])) AND (Sport[Title/abstract] OR sport*[Title/abstract] OR athlete*[Title/abstract])) AND (female[Title/abstract] OR women*[Title/abstract] OR girl*[Title/abstract] OR gender*[Title/abstract])) NOT ("case report"[Title/abstract] OR review[Title/abstract])
Search Date: October 23, 2023
*EMBASE search:*
EMBASE OVID <1974 to 2023 October 12>
1 (injuries or injur$ or Trauma or Fracture or Sprain or traumatic or acute).ti,ab.
2 (foot or Ankle or Toe or lower extremity or lower extremit$ or lower limb or lower body or Lisfranc$ or metatarsal$ or tarsal or Ankle or hindfoot or heel or achilles$).ti,ab.
3 (Sport or sport$ or athlete$).ti,ab.
4 (female or women$ or girl$ or gender$).ti,ab.
5 ("case report" or review).ti,ab.
6 (1 and 2 and 3 and 4) not 5
Date of Search: October 23, 2023
Results: 2115
*Medline search:*
Ovid MEDLINE(R) ALL <1946 to October 12, 2023>
1 (injuries or injur$ or Trauma or Fracture or Sprain or traumatic or acute).ti,ab.
2 (foot or Ankle or Toe or lower extremity or lower extremit$ or lower limb or lower body or Lisfranc$ or metatarsal$ or tarsal or Ankle or hindfoot or heel or achilles$).ti,ab.
3 (Sport or sport$ or athlete$).ti,ab.
4 (female or women$ or girl$ or gender$).ti,ab.
5 ("case report" or review).ti,ab.
6 (1 and 2 and 3 and 4) not 5
Date of Search: October 23, 2023

**Appendix Table A2:** Final Studies and Data Included in Meta-Analyses^*a*^

Study	No. Injured, Female	Total N, Female	No. Injured, Male	Total N, Male	Unit of Measure	Level of Competition	Sport	Subgroup*^*b*^*
Adachi et al, 2022^1^	16	5423	13	5892	abs	Professional	Summer Olympics	1
Badekas et al, 2009^7^	201	266	324	358	abs	Professional	Summer Olympics	1
Edouard et al, 2018^26^	30	625	4	338	abs	Professional	Gymnastics	1
Kirialanis et al, 2003^44^	40	79	29	83	abs	Professional	Gymnastics	1
Langevoort et al, 2007^48^	103	161	94	132	abs	Professional	Handball	1
Manaf et al, 2021^56^	15	41	9	43	abs	Professional	Field hockey	1
Mateos Conde et al, 2022^57^	30	34	43	70	abs	Professional	Basketball	1
Neidel et al, 2019^61^	12	39	8	47	abs	Professional	Triathlon	1
Rice et al, 2022^73^	45	126	40	111	abs	Professional	Tennis	1
Ruddick et al, 2019^83^	49	66	28	86	abs	Professional	Basketball, netball, hockey, football, swimming	1
Snellman et al, 2001^87^	14	96	19	199	abs	Professional	Football	1
Tranaeus et al, 2016^90^	39	116	22	122	abs	Professional	Floorball	1
Beneka et al, 2007^10^	36	151	40	159	abs	Semiprofessional	Volleyball	2
Hosea et al, 2000^36^	70	364	78	504	abs	Semiprofessional	Basketball	2
Hunt et al, 2017^37^	124	496	104	580	abs	Semiprofessional	Multiple	2
Kay et al, 2017^39^	227	765	209	957	abs	Semiprofessional	Basketball, cross-country, football, softball, tennis, volleyball, lacrosse, football, swimming, tennis, track and field	2
Kelley et al, 2023^40^	77	159	60	119	abs	Semiprofessional	Basketball, cross-country, football, softball, tennis, volleyball	2
Roos et al, 2015^80^	658	1703	556	1866	abs	Semiprofessional	Baseball/softball, basketball, cross-country, football, swimming, tennis, track and field	2
Rosa et al, 2014^82^	19	127	17	165	abs	Semiprofessional	Basketball, track and field	2
Sallis et al, 2001^84^	856	1874	1018	1893	abs	Semiprofessional	Basketball, track and field, swimming, soccer, tennis, water polo, cross-country	2
Wayner et al, 2023^98^	116	714	52	506	abs	Semiprofessional	Cross-country	2
Alonso et al, 2009^3^	22	1340	18	2009	AE	Professional	Track and field	3
Bere et al, 2015^11^	77	13485	82	12148	AE	Professional	Volleyball	3
Deitch et al, 2006^22^	470	22980	931	70420	AE	Professional	Basketball	3
Edouard et al, 2015^25^	308	6930	450	8135	AE	Professional	Track and field	3
Ekstrand et al, 2011^27^	57	55456	294	218265	AE	Professional	Football	3
Hagglund et al, 2009^33^	110	54156	153	71361	AE	Professional	Football	3
Junge et al, 2006^38^	33	1770	61	5706	AE	Professional	Football, handball, basketball, field hockey, baseball, softball, water polo, volleyball	3
Larruskain et al, 2018^49^	47	50788	44	77756	AE	Professional	Football	3
Pasanen et al, 2016^67^	7	1263	13	1316	AE	Professional	Floorball	3
Roh et al, 2022^78^	85	70216	70	75386	AE	Professional	Handball	3
Vanlommel et al, 2013^93^	647	21684	953	102362	AE	Professional	Football	3
Verhagen et al, 2004^94^	56	23245	44	14867	AE	Professional	Volleyball	3
Baugh et al, 2018^9^	149	72180	15	19913	AE	Semiprofessional	Volleyball	4
Beynnon et al, 2005^12^	29	7802	14	6404	AE	Semiprofessional	Lacrosse, football, hockey, basketball	4
Chan et al, 2020^14^	83	4345128	172	7433137	AE	Semiprofessional	Basketball, cross-country, soccer, tennis, track and field	4
Chandran et al, 2023^15^	956	2569448	1052	2637630	AE	Semiprofessional	Basketball, cross-country, lacrosse, soccer, swimming, tennis, track and field	4
Crowley et al, 2019^21^	103	231928	413	552623	AE	Semiprofessional	Ice hockey	4
Gulbrandsen et al, 2019^32^	1084	781948	984	677238	AE	Semiprofessional	Football	4
Hootman et al, 2007^35^	7861	2097635	9327	3146453	AE	Semiprofessional	Basketball, lacrosse, football, ice hockey	4
Kerr et al, 2008^41^	63	24931	47	48903	AE	Semiprofessional	Rugby union	4
Kerr et al, 2016^43^	114	44472	138	46332	AE	Semiprofessional	Cross-country	4
Kopec et al, 2017^46^	97	1515625	105	1779661	AE	Semiprofessional	Basketball, cross-country, football, softball, tennis, volleyball, lacrosse, football, swimming, tennis, track and field	4
Lievers et al, 2020^52^	766	1814509	1201	3007476	AE	Semiprofessional	Basketball, cross-country, ice hockey, lacrosse, football, swimming, tennis, track and field	4
Lynall et al, 2016^54^	73	46530	44	36994	AE	Semiprofessional	Tennis	4
Lytle et al, 2021^55^	653	245072	483	367608	AE	Semiprofessional	Basketball, gymnastics, volleyball	4
Owoeye et al, 2017^65^	14	759	8	1023	AE	Semiprofessional	Football	4
Rizzone et al, 2017^75^	175	3493450	107	4054457	AE	Semiprofessional	Baseball/softball, basketball, cross-country, ice hockey, lacrosse, football, swimming, tennis, track and field	4
Roos et al, 2017^81^	518	269112	506	192538	AE	Semiprofessional	Football	4
Roos et al, 2017^79^	672	717949	756	972972	AE	Semiprofessional	Baseball/softball, basketball, cross-country, football, swimming, tennis, track and field	4
Schick and Meeuwisse, 2003^85^	8	4028	8	5195	AE	Semiprofessional	Ice hockey	4
Westermann et al, 2015^99^	201	21453	240	27348	AE	Semiprofessional	Gymnastics	4
Zuckerman et al, 2018^103^	585	249506	745	289406	AE	Semiprofessional	Basketball	4

a For the purposes of analysis, we included professional athletes as those participating at national level or international level (eg, premier division football or Olympic Games) and semiprofessional as below this level (eg, American collegiate athletes). abs, absolute athlete number; AE, athletic exposure.

b Subgroups: 1 = professional reporting absolute numbers; 2 = semiprofessional reporting absolute numbers; 3 = professional reporting exposures; 4 = semiprofessional reporting exposures.

## References

[1] Adachi T Katagiri H An JS , et al. Imaging-detected bone stress injuries at the Tokyo 2020 summer Olympics: epidemiology, injury onset, and competition withdrawal rate. BMC Musculoskelet Disord. 2022;23(1):763. doi:10.1186/s12891-022-05725-8 35948918 PMC9364573

[2] Adgate B . Popularity of women’s sports surges approaching 50th anniversary of Title IX. Forbes.com . Accessed October 22, 2023. https://www.forbes.com/sites/bradadgate/2022/04/07/popularity-of-womens-sports-has-been-surging/

[3] Alonso JM Junge A Renström P Engebretsen L Mountjoy M Dvorak J . Sports injuries surveillance during the 2007 IAAF World Athletics Championships. Clin J Sport Med. 2009;19(1):26-32. doi:10.1097/jsm.0b013e318191c8e7 19124980

[4] Anderson T Wasserman EB Shultz SJ . Anterior cruciate ligament injury risk by season period and competition segment: an analysis of National Collegiate Athletic Association injury surveillance data. J Athl Train. 2019;54(7):787-795. doi:10.4085/1062-6050-501-17 31322904 PMC6709760

[5] Ardern CL Büttner F Andrade R , et al. Implementing the 27 PRISMA 2020 Statement items for systematic reviews in the sport and exercise medicine, musculoskeletal rehabilitation and sports science fields: the PERSiST (implementing Prisma in Exercise, Rehabilitation, Sport medicine and SporTs science) guidance. Br J Sports Med. 2022;56(4):175-195. doi:10.1136/bjsports-2021-103987 34625401 PMC8862073

[6] Ashizawa K Kumakura C Kusumoto A Narasaki S . Relative foot size and shape to general body size in Javanese, Filipinas and Japanese with special reference to habitual footwear types. Ann Hum Biol. 1997;24(2):117-129. doi:10.1080/03014469700004862 9074748

[7] Badekas T Papadakis SA Vergados N , et al. Foot and ankle injuries during the Athens 2004 Olympic Games. J Foot Ankle Res. 2009;2:9. doi:10.1186/1757-1146-2-9 19361341 PMC2672073

[8] Baker H Rizzi A Athiviraham A . Injury in the Women’s National Basketball Association (WNBA) from 2015 to 2019. Arthrosc Sports Med Rehabil. 2020;2(3):e213-e217. doi:10.1016/j.asmr.2020.02.003 PMC7283941 32548586

[9] Baugh CM Weintraub GS Gregory AJ Djoko A Dompier TP Kerr ZY . Descriptive epidemiology of injuries sustained in National Collegiate Athletic Association men’s and women’s volleyball, 2013-2014 to 2014-2015. Sports Health. 2018;10(1):60-69. doi:10.1177/1941738117733685 28985702 PMC5753967

[10] Beneka A Malliou P Tsigganos G , et al. A prospective study of injury incidence among elite and local division volleyball players in Greece. J Back Musculoskelet Rehabil. 2007;20(2-3):115-121. doi:10.3233/bmr-2007-202-309

[11] Bere T Kruczynski J Veintimilla N Hamu Y Bahr R . Injury risk is low among world-class volleyball players: 4-year data from the FIVB Injury Surveillance System. Br J Sports Med. 2015;49(17):1132-1137. doi:10.1136/bjsports-2015-094959 26194501 PMC4552924

[12] Beynnon BD Vacek PM Murphy D Alosa D Paller D . First-time inversion ankle ligament trauma: the effects of sex, level of competition, and sport on the incidence of injury. Am J Sports Med. 2005;33(10):1485-1491. doi:10.1177/0363546505275490 16009979

[13] Bretzin AC D’Alonzo BA Chandran A , et al. Epidemiology of injuries in National Collegiate Athletic Association women’s lacrosse: 2014-2015 through 2018-2019. J Athl Train. 2021;56(7):750-757. doi:10.4085/1062-6050-613-20 34280267 PMC8293888

[14] Chan JJ Chen KK Sarker S , et al. Epidemiology of Achilles tendon injuries in collegiate level athletes in the United States. Int Orthop. 2020;44(3):585-594. doi:10.1007/s00264-019-04471-2 31907586

[15] Chandran A Moffit RE DeJong Lempke AF , et al. Epidemiology of lateral ligament complex tears of the ankle in National Collegiate Athletic Association (NCAA) sports: 2014-15 through 2018-19. Am J Sports Med. 2023;51(1):169-178. doi:10.1177/03635465221138281 36592020

[16] Chandran A Morris SN Boltz AJ Robison HJ Collins CL . Epidemiology of injuries in National Collegiate Athletic Association women’s cross-country: 2014-2015 through 2018-2019. J Athl Train. 2021;56(7):622-628. doi:10.4085/1062-6050-395-20 34280273 PMC8293874

[17] Chandran A Morris SN Boltz AJ Robison HJ Collins CL . Epidemiology of injuries in National Collegiate Athletic Association women’s soccer: 2014-2015 through 2018-2019. J Athl Train. 2021;56(7):651-658. doi:10.4085/1062-6050-372-20 34280264 PMC8293894

[18] Chandran A Morris SN Lempke LB Boltz AJ Robison HJ Collins CL . Epidemiology of injuries in National Collegiate Athletic Association women’s volleyball: 2014-2015 through 2018-2019. J Athl Train. 2021;56(7):666-673. doi:10.4085/1062-6050-679-20 34280268 PMC8293869

[19] Chandrashekar N Slauterbeck J Hashemi J . Sex-based differences in the anthropometric characteristics of the anterior cruciate ligament and its relation to intercondylar notch geometry: a cadaveric study. Am J Sports Med. 2005;33(10):1492-1498. doi:10.1177/0363546504274149 16009992

[20] Collings TJ Bourne MN Barrett RS Du Moulin W Hickey JT Diamond LE . Risk factors for lower limb injury in female team field and court sports: a systematic review, meta-analysis, and best evidence synthesis. Sports Med. 2021;51(4):759-776. doi:10.1007/s40279-020-01410-9 33400215

[21] Crowley SG Trofa DP Vosseller JT , et al. Epidemiology of foot and ankle injuries in National Collegiate Athletic Association men’s and women’s ice hockey. Orthop J Sports Med. 2019;7(8):2325967119865908. doi:10.1177/2325967119865908 PMC6713968 31489332

[22] Deitch JR Starkey C Walters SL Moseley JB . Injury risk in professional basketball players: a comparison of Women’s National Basketball Association and National Basketball Association athletes. Am J Sports Med. 2006;34(7):1077-1083. doi:10.1177/0363546505285383 16493173

[23] DiStefano LJ Dann CL Chang CJ , et al. The first decade of web-based sports injury surveillance: descriptive epidemiology of injuries in US high school girls’ soccer (2005-2006 through 2013-2014) and National Collegiate Athletic Association women’s soccer (2004-2005 through 2013-2014). J Athl Train. 2018;53(9):880-892. doi:10.4085/1062-6050-156-17 30372637 PMC6208306

[24] Doherty C Delahunt E Caulfield B Hertel J Ryan J Bleakley C . The incidence and prevalence of ankle sprain injury: a systematic review and meta-analysis of prospective epidemiological studies. Sports Med. 2014;44(1):123-140. doi:10.1007/s40279-013-0102-5 24105612

[25] Edouard P Feddermann-Demont N Alonso JM Branco P Junge A . Sex differences in injury during top-level international athletics championships: surveillance data from 14 championships between 2007 and 2014. Br J Sports Med. 2015;49(7):472-477. doi:10.1136/bjsports-2014-094316 25618889

[26] Edouard P Steffen K Junge A Leglise M Soligard T Engebretsen L . Gymnastics injury incidence during the 2008, 2012 and 2016 Olympic Games: analysis of prospectively collected surveillance data from 963 registered gymnasts during Olympic Games. Br J Sports Med. 2018;52(7):475-481. doi:10.1136/bjsports-2017-097972 29032364

[27] Ekstrand J Hägglund M Fuller CW . Comparison of injuries sustained on artificial turf and grass by male and female elite football players. Scand J Med Sci Sports. 2011;21(6):824-832. doi:10.1111/j.1600-0838.2010.01118.x 20456680

[28] Ericksen H Gribble PA . Sex differences, hormone fluctuations, ankle stability, and dynamic postural control. J Athl Train. 2012;47(2):143-148. doi:10.4085/1062-6050-47.2.143 22488279 PMC3418125

[29] Finch CF Kemp JL Clapperton AJ . The incidence and burden of hospital-treated sports-related injury in people aged 15+ years in Victoria, Australia, 2004-2010: a future epidemic of osteoarthritis? Osteoarthritis Cartilage. 2015;23(7):1138-1143. doi:10.1016/j.joca.2015.02.165 25749009

[30] Fuller CW . Injury risk (burden), risk matrices and risk contours in team sports: a review of principles, practices and problems. Sports Med. 2018;48(7):1597-1606. doi:10.1007/s40279-018-0913-5 29623603

[31] Gianakos AL George N Merklein M , et al. Foot and ankle related sex-specific analysis within high-impact journals. Foot Ankle Int. 2020;41(3):356-363. doi:10.1177/1071100719894530 31855079

[32] Gulbrandsen M Hartigan DE Patel KA Makovicka JL Tummala SV Chhabra A . Ten-year epidemiology of ankle injuries in men’s and women’s collegiate soccer players. J Athl Train. 2019;54(8):881-888. doi:10.4085/1062-6050-144-18 31390272 PMC6756601

[33] Hägglund M Waldén M Ekstrand J . Injuries among male and female elite football players. Scand J Med Sci Sports. 2009;19(6):819-827. doi:10.1111/j.1600-0838.2008.00861.x 18980604

[34] Higgins JPT Morgan RL Rooney AA , et al. A tool to assess risk of bias in non-randomized follow-up studies of exposure effects (ROBINS-E). Environ Int. 2024;186:108602. doi:10.1016/j.envint.2024.108602 38555664 PMC11098530

[35] Hootman JM Dick R Agel J . Epidemiology of collegiate injuries for 15 sports: summary and recommendations for injury prevention initiatives. J Athl Train. 2007;42(2):311-319. 17710181 PMC1941297

[36] Hosea TM Carey CC Harrer MF . The gender issue: epidemiology of ankle injuries in athletes who participate in basketball. Clin Orthop Relat Res. 2000;372:45-49. doi:10.1097/00003086-200003000-00006 10738413

[37] Hunt KJ Hurwit D Robell K Gatewood C Botser IB Matheson G . Incidence and epidemiology of foot and ankle injuries in elite collegiate athletes. Am J Sports Med. 2017;45(2):426-433. doi:10.1177/0363546516666815 27802962

[38] Junge A Langevoort G Pipe A , et al. Injuries in team sport tournaments during the 2004 Olympic Games. Am J Sports Med. 2006;34(4):565-576. doi:10.1177/0363546505281807 16303876

[39] Kay MC Register-Mihalik JK Gray AD Djoko A Dompier TP Kerr ZY . The epidemiology of severe injuries sustained by National Collegiate Athletic Association student-athletes, 2009-2010 through 2014-2015. J Athl Train. 2017;52(2):117-128. doi:10.4085/1062-6050-52.1.01 28118030 PMC5343524

[40] Kelley EA Hogg JA Gao L Waxman JP Shultz SJ . Demographic factors and instantaneous lower extremity injury occurrence in a National Collegiate Athletic Association Division I population. J Athl Train. 2023;58(5):393-400. doi:10.4085/1062-6050-0673.21 35789230 PMC11220903

[41] Kerr HA Curtis C Micheli LJ , et al. Collegiate rugby union injury patterns in New England: a prospective cohort study. Br J Sports Med. 2008;42(7):595-603. doi:10.1136/bjsm.2007.035881 18203866

[42] Kerr ZY Hayden R Barr M Klossner DA Dompier TP . Epidemiology of National Collegiate Athletic Association women’s gymnastics injuries, 2009-2010 through 2013-2014. J Athl Train. 2015;50(8):870-878. doi:10.4085/1062-6050-50.7.02 26196702 PMC4629945

[43] Kerr ZY Kroshus E Grant J , et al. Epidemiology of National Collegiate Athletic Association men’s and women’s cross-country injuries, 2009-2010 through 2013-2014. J Athl Train. 2016;51(1):57-64. doi:10.4085/1062-6050-51.1.10 26701643 PMC4851130

[44] Kirialanis P Malliou P Beneka A Giannakopoulos K . Occurrence of acute lower limb injuries in artistic gymnasts in relation to event and exercise phase. Br J Sports Med. 2003;37(2):137-139. doi:10.1136/bjsm.37.2.137 12663355 PMC1724619

[45] Kooy CEVW Jakobsen RB Fenstad AM , et al. Major increase in incidence of pediatric ACL reconstructions from 2005 to 2021: a study from the Norwegian Knee Ligament Register. Am J Sports Med. 2023;51(11):2891-2899. doi:10.1177/03635465231185742 37497771 PMC10478322

[46] Kopec TJ Hibberd EE Roos KG Djoko A Dompier TP Kerr ZY . The epidemiology of deltoid ligament sprains in 25 National Collegiate Athletic Association sports, 2009-2010 through 2014-2015 academic years. J Athl Train. 2017;52(4):350-359. doi:10.4085/1062.6050-52.2.01 28318315 PMC5402533

[47] Kubo K Kanehisa H Fukunaga T . Gender differences in the viscoelastic properties of tendon structures. Eur J Appl Physiol. 2003;88(6):520-526. doi:10.1007/s00421-002-0744-8 12560950

[48] Langevoort G Myklebust G Dvorak J Junge A . Handball injuries during major international tournaments. Scand J Med Sci Sports. 2007;17(4):400-407. doi:10.1111/j.1600-0838.2006.00587.x 17038157

[49] Larruskain J Lekue JA Diaz N Odriozola A Gil SM . A comparison of injuries in elite male and female football players: a five-season prospective study. Scand J Med Sci Sports. 2018;28(1):237-245. doi:10.1111/sms.12860 28207979

[50] Lempke LB Chandran A Boltz AJ Robison HJ Collins CL Morris SN . Epidemiology of injuries in National Collegiate Athletic Association women’s basketball: 2014-2015 through 2018-2019. J Athl Train. 2021;56(7):674-680. doi:10.4085/1062-6050-466-20 34280270 PMC8293880

[51] Li G Boncz I Járomi M Molics B Ács P Tardi P . SA70 survey of sport-specific lower limb injuries in 15 of Hungary’s most popular sports. Value Health. 2022;25(12):S496-S497. doi:10.1016/j.jval.2022.09.2463

[52] Lievers WB Goggins KA Adamic P . Epidemiology of foot injuries using National Collegiate Athletic Association data from the 2009-2010 through 2014-2015 seasons. J Athl Train. 2020;55(2):181-187. doi:10.4085/1062-6050-560-18 31895592 PMC7017902

[53] Lohmander LS Ostenberg A Englund M Roos H . High prevalence of knee osteoarthritis, pain, and functional limitations in female soccer players twelve years after anterior cruciate ligament injury. Arthritis Rheum. 2004;50(10):3145-3152. doi:10.1002/art.20589 15476248

[54] Lynall RC Kerr ZY Djoko A Pluim BM Hainline B Dompier TP . Epidemiology of National Collegiate Athletic Association men’s and women’s tennis injuries, 2009/2010-2014/2015. Br J Sports Med. 2016;50(19):1211-1216. doi:10.1136/bjsports-2015-095360 26719502

[55] Lytle JB Parikh KB Tarakemeh A Vopat BG Mulcahey MK . Epidemiology of foot and ankle injuries in NCAA jumping athletes in the United States during 2009-2014. Orthop J Sports Med. 2021;9(4):2325967121998052. doi:10.1177/2325967121998052 PMC8053761 33948444

[56] Manaf H Justine M Hassan N . Prevalence and pattern of musculoskeletal injuries among Malaysian Hockey League players. Malays Orthop J. 2021;15(1):21-26. doi:10.5704/moj.2103.004 PMC8043635 33880144

[57] Mateos Conde J Cabero Morán MT Moreno Pascual C . Prospective epidemiological study of basketball injuries during one competitive season in professional and amateur Spanish basketball. Phys Sportsmed. 2022;50(4):349-358. doi:10.1080/00913847.2021.1943721 34151718

[58] McGuinness LA Higgins JPT . Risk-of-bias VISualization (robvis): an R package and shiny web app for visualizing risk-of-bias assessments. Res Synth Methods. 2021;12(1):55-61. doi:10.1002/jrsm.1411 32336025

[59] Mendiguchia J Ford KR Quatman CE Alentorn-Geli E Hewett TE . Sex differences in proximal control of the knee joint. Sports Med. 2011;41(7):541-557. doi:10.2165/11589140-000000000-00000 21688868 PMC4221796

[60] Montalvo AM Schneider DK Yut L , et al. “What’s my risk of sustaining an ACL injury while playing sports?” A systematic review with meta-analysis. Br J Sports Med. 2019;53(16):1003-1012. doi:10.1136/bjsports-2016-096274 29514822 PMC6561829

[61] Neidel P Wolfram P Hotfiel T , et al. Cross-sectional investigation of stress fractures in German elite triathletes. Sports (Basel). 2019;7(4):88. doi:10.3390/sports7040088 30991761 PMC6524354

[62] O’Connor AM James IT . Association of lower limb injury with boot cleat design and playing surface in elite soccer. Foot Ankle Clin. 2013;18(2):369-380. doi:10.1016/j.fcl.2013.02.012 23707183

[63] O’Connor K Bragdon G Baumhauer JF . Sexual dimorphism of the foot and ankle. Orthop Clin North Am. 2006;37(4):569-574. doi:10.1016/j.ocl.2006.09.008 17141014

[64] Okholm Kryger K Thomson A Tang A , et al. Ten questions in sports engineering: technology in elite women’s football. Sports Eng. 2022;25(1):25. doi:10.1007/s12283-022-00384-3

[65] Owoeye OBA Aiyegbusi AI Fapojuwo OA Badru OA Babalola AR . Injuries in male and female semi-professional football (soccer) players in Nigeria: prospective study of a National Tournament. BMC Res Notes. 2017;10(1):133. doi:10.1186/s13104-017-2451-x 28327163 PMC5361784

[66] Page MJ McKenzie JE Bossuyt PM , et al. The PRISMA 2020 statement: an updated guideline for reporting systematic reviews. BMJ. 2021;372:N71. doi:10.1136/bmj.n71 PMC8005924 33782057

[67] Pasanen K Bruun M Vasankari T Nurminen M Frey WO . Injuries during the international floorball tournaments from 2012 to 2015. BMJ Open Sport Exerc Med. 2016;2(1):e000217. doi:10.1136/bmjsem-2016-000217 PMC5566261 28890804

[68] Patel N Bhatia A Mullen C Bosman E Lear A . Professional women’s softball injuries: an epidemiological cohort study. Clin J Sport Med. 2021;31(1):63-69. doi:10.1097/JSM.0000000000000698 31233431

[69] Polycarpou V . The Rise of Female Sports. Sports Financial Literacy Academy. March 2, 2022. Accessed October 22, 2023. https://moneysmartathlete.com/women-athletes/the-rise-of-female-sports/

[70] Prien A Grafe A Rössler R Junge A Verhagen E . Epidemiology of head injuries focusing on concussions in team contact sports: a systematic review. Sports Med. 2018;48(4):953-969. doi:10.1007/s40279-017-0854-4 29349651

[71] Quatman CE Ford KR Myer GD Paterno MV Hewett TE . The effects of gender and pubertal status on generalized joint laxity in young athletes. J Sci Med Sport. 2008;11(3):257-263. doi:10.1016/j.jsams.2007.05.005 17597005 PMC2453596

[72] Quintana-Cepedal M Rodríguez MÁ Crespo I , et al. Injury characteristics among young adults during and immediately after the COVID-19 lockdown. Int J Environ Res Public Health. 2022;19(15):8982. doi:10.3390/ijerph19158982 35897353 PMC9329858

[73] Rice RP Roach K Kirk-Sanchez N , et al. Age and gender differences in injuries and risk factors in elite junior and professional tennis players. Sports Health. 2022;14(4)466-477. doi:10.1177/19417381211062834 35037501 PMC9214903

[74] Risk of Bias Tools. robvis (visualization tool). Riskofbias.info. Accessed June 23, 2024. https://www.riskofbias.info/welcome/robvis-visualization-tool

[75] Rizzone KH Ackerman KE Roos KG Dompier TP Kerr ZY . The epidemiology of stress fractures in collegiate student-athletes, 2004-2005 through 2013-2014 academic years. J Athl Train. 2017;52(10):966-975. doi:10.4085/1062-6050-52.8.01 28937802 PMC5687241

[76] Robison HJ Boltz AJ Morris SN Collins CL Chandran A . Epidemiology of injuries in National Collegiate Athletic Association women’s tennis: 2014-2015 through 2018-2019. J Athl Train. 2021;56(7):766-772. doi:10.4085/1062-6050-529-20 34280290 PMC8293885

[77] Rodrigues P Chang R TenBroek T Van Emmerik R Hamill J . Evaluating the coupling between foot pronation and tibial internal rotation continuously using vector coding. J Appl Biomech. 2015;31(2):88-94. doi:10.1123/JAB.2014-0067 25386828

[78] Roh HL Kim CW Park KJ . Epidemiology of injuries in elite Korean handball athletes: a prospective cohort study. J Sports Med Phys Fitness. 2022;62(1):90-97. doi:10.23736/s0022-4707.21.12121-8 33615763

[79] Roos KG Kerr ZY Mauntel TC Djoko A Dompier TP Wikstrom EA . The Epidemiology of lateral ligament complex ankle sprains in National Collegiate Athletic Association sports. Am J Sports Med. 2017;45(1):201-209. doi:10.1177/0363546516660980 27573356

[80] Roos KG Marshall SW Kerr ZY , et al. Epidemiology of overuse injuries in collegiate and high school athletics in the United States. Am J Sports Med. 2015;43(7):1790-1797. doi:10.1177/0363546515580790 25930673

[81] Roos KG Wasserman EB Dalton SL , et al. Epidemiology of 3825 injuries sustained in six seasons of National Collegiate Athletic Association men’s and women’s soccer (2009/2010-2014/2015). Br J Sports Med. 2017;51(13):1029-1034. doi:10.1136/bjsports-2015-095718 27190140

[82] Rosa BB Asperti AM Helito CP Demange MK Fernandes TL Hernandez AJ . Epidemiology of sports injuries on collegiate athletes at a single center. Acta Ortop Bras. 2014;22(6):321-324. doi:10.1590/1413-78522014220601007 25538479 PMC4273958

[83] Ruddick GK Lovell GA Drew MK Fallon KE . Epidemiology of bone stress injuries in Australian high performance athletes: a retrospective cohort study. J Sci Med Sport. 2019;22(10):1114-1118. doi:10.1016/j.jsams.2019.06.008 31307905

[84] Sallis RE Jones K Sunshine S Smith G Simon L . Comparing sports injuries in men and women. Int J Sports Med. 2001;22(6):420-423. doi:10.1055/s-2001-16246 11531034

[85] Schick DM Meeuwisse WH . Injury rates and profiles in female ice hockey players. Am J Sports Med. 2003;31(1):47-52. doi:10.1177/03635465030310011901 12531756

[86] Shaffer RA Brodine SK Ito SI Le AT . Epidemiology of illness and injury among U.S. Navy and Marine Corps female training populations. Mil Med. 1999;164(1):17-21. 9922638

[87] Snellman K Parkkari J Kannus P Leppälä J Vuori I Järvinen M . Sports injuries in floorball: a prospective one-year follow-up study. Int J Sports Med. 2001;22(7):531-536. doi:10.1055/s-2001-17609 11590481

[88] Stroup DF . Meta-analysis of observational studies in epidemiology: a proposal for reporting. JAMA. 2000;283(15):2008. doi:10.1001/jama.283.15.2008 10789670

[89] Tanaka S Sagisaka R Sone E Tanaka H . Sport level and sex differences in sport-related concussion among Japanese collegiate athletes: epidemiology, knowledge, reporting behaviors, and reported symptoms. Sports Med Health Sci. 2023;5(3):229-238. doi:10.1016/j.smhs.2023.07.002 37753424 PMC10518792

[90] Tranaeus U Götesson E Werner S . Injury profile in Swedish elite floorball. Sports Health. 2016;8(3):224-229. doi:10.1177/1941738116628472 26823181 PMC4981064

[91] Tricco AC Lillie E Zarin W , et al. PRISMA extension for scoping reviews (PRISMA-ScR): checklist and explanation. Ann Intern Med. 2018;169(7):467-473. doi:10.7326/M18-0850 30178033

[92] Trost SG Pate RR Sallis JF , et al. Age and gender differences in objectively measured physical activity in youth. Med Sci Sports Exerc. 2002;34(2):350-355. doi:10.1097/00005768-200202000-00025 11828247

[93] Vanlommel L Vanlommel J Bollars P , et al. Incidence and risk factors of lower leg fractures in Belgian soccer players. Injury. 2013;44(12):1847-1850. doi:10.1016/j.injury.2013.07.002 23916900

[94] Verhagen EALM Van der Beek AJ Bouter LM Bahr RM Van Mechelen W . A one season prospective cohort study of volleyball injuries. Br J Sports Med. 2004;38(4):477-481. doi:10.1136/bjsm.2003.005785 15273190 PMC1724865

[95] Viechtbauer W . Bias and efficiency of meta-analytic variance estimators in the random-effects model. J Educ Behav Stat. 2005;30(3):261-293. doi:10.3102/10769986030003261

[96] Waldén M Hägglund M Ekstrand J . Football injuries during European Championships 2004-2005. Knee Surg Sports Traumatol Arthrosc. 2007;15(9):1155-1162. doi:10.1007/s00167-007-0290-3 17375283

[97] Warden SJ Creaby MW Bryant AL Crossley KM . Stress fracture risk factors in female football players and their clinical implications. Br J Sports Med. 2007;41(suppl 1):i38-i43. doi:10.1136/bjsm.2007.037804 PMC2465247 17584950

[98] Wayner RA Brown CN Bovbjerg VE , et al. Epidemiology of bone stress injuries and healthcare utilization in PAC-12 cross-country athletes. J Athl Train. 2024;59(6):641-648. doi:10.4085/1062-6050-0089.23 37459389 PMC11220775

[99] Westermann RW Giblin M Vaske A Grosso K Wolf BR . Evaluation of men’s and women’s gymnastics injuries: a 10-year observational study. Sports Health. 2015;7(2):161-165. doi:10.1177/1941738114559705 25984262 PMC4332645

[100] Wilkerson RD Mason MA . Differences in men’s and women’s mean ankle ligamentous laxity. Iowa Orthop J. 2000;20:46-48. 10934624 PMC1888743

[101] Wunderlich RE Cavanagh PR . Gender differences in adult foot shape: implications for shoe design: Med Sci Sports Exerc. 2001;33(4):605-611. doi:10.1097/00005768-200104000-00015 11283437

[102] Zech A Hollander K Junge A , et al. Sex differences in injury rates in team-sport athletes: a systematic review and meta-regression analysis. J Sport Health Sci. 2022;11(1):104-114. doi:10.1016/j.jshs.2021.04.003 34052518 PMC8847930

[103] Zuckerman SL Wegner AM Roos KG Djoko A Dompier TP Kerr ZY . Injuries sustained in National Collegiate Athletic Association men’s and women’s basketball, 2009/2010-2014/2015. Br J Sports Med. 2018;52(4):261-268. doi:10.1136/bjsports-2016-096005 27364907

